# Impact of Excluding Anti‐HLA‐C and ‐DP Antibodies From the Allocation System on Kidney Transplant Access

**DOI:** 10.1111/tan.70822

**Published:** 2026-07-07

**Authors:** Margot Lepage, Thomas Barba, Xavier Charmetant, Luc Chauvelot, Manon Guezennec, Kevin Goret‐Roycourt, Fanny Buron, Olivier Thaunat, Emmanuel Morelon, Valérie Dubois, Alice Koenig

**Affiliations:** ^1^ National Blood Service (EFS), HLA Laboratory Décines‐Charpieu France; ^2^ Department of Internal Medicine Edouard Herriot Hospital, Hospices Civils de Lyon Lyon France; ^3^ CIRI, INSERM U1111, Université Claude Bernard Lyon I, CNRS UMR5308, Ecole Normale Supérieure de Lyon, Univ. Lyon Lyon France; ^4^ Department of Transplantation, Nephrology and Clinical Immunology Edouard Herriot Hospital, Hospices Civils de Lyon Lyon France; ^5^ Lyon‐Est Medical Faculty Claude Bernard University (Lyon 1) Lyon France

**Keywords:** anti‐HLA‐C antibodies, anti‐HLA‐DP antibodies, donor specific antibodies, graft access, graft allocation, kidney transplantation, panel reactive antibody, unacceptable antigens

## Abstract

Given the risk of antibody‐mediated rejection, most kidney graft allocation systems define unacceptable antigens to prevent transplantation in the presence of donor‐specific antibodies. Anti‐HLA‐C and anti‐HLA‐DP antibodies are often not included in these algorithms, including the French allocation system, despite growing evidence supporting their pathogenicity. In routine clinical practice, however, these antibodies are frequently considered by transplant teams when evaluating donor offers, creating a discrepancy between allocation scores and real‐world access to transplantation. We aimed to evaluate the impact of this discrepancy on access to kidney transplantation. We conducted a retrospective study including 2160 patients registered on the kidney transplant waiting list at our centre between 2009 and 2018, in whom anti‐HLA‐C and anti‐HLA‐DP antibodies were systematically considered in clinical decision‐making similarly to other HLA antibodies. Anti‐HLA‐C/DP antibodies were detected in 325 patients (15.1%). Incorporating these antibodies into cPRA calculations resulted in a near‐systematic increase in cPRA values (mean increase, 5.5 ± 11.3). Using relative loss of graft access (RLGA), 83.5% of patients experienced reduced estimated access to transplantation, including 21.5% who lost more than 75% of their access. Patients harbouring anti‐HLA‐C/DP antibodies also exhibited significantly reduced access to kidney transplantation in both univariable and multivariable analyses (HR = 0.52; 95% CI, 0.42–0.66; *p* = 2 × 10^−8^), associated with higher immunological donor offer refusal rates (mean 25.7% vs. 1.8%, *p* < 2 × 10^−16^). In conclusion, excluding anti‐HLA‐C and anti‐HLA‐DP antibodies from allocation algorithms, despite their consideration in routine clinical decision‐making, is associated with reduced access to kidney transplantation, and their integration could improve equity among transplant candidates.

## 1 Introduction

Kidney graft allocation relies on a combination of clinical, immunological, geographical, and demographic criteria, designed to balance the principles of utility and equity [1]. Among these determinants, immunological compatibility between donors and recipients plays a pivotal role, as it directly influences both graft outcomes and access to transplantation [2].

To reduce the risk of antibody‐mediated rejection, allocation systems define for each candidate a list of unacceptable antigens corresponding to HLA specificities against which the recipient has preformed antibodies. The integration of these unacceptable antigens into allocation algorithms has led to the widespread use of the calculated panel reactive antibody (cPRA), which estimates the proportion of incompatible donors for a given patient and is now a cornerstone metric for prioritising highly sensitised candidates. In the French allocation system, candidates with a cPRA ≥ 85% are classified as hyperimmunised and benefit from increased allocation priority. Historically, most allocation systems have focused on antibodies directed against HLA‐A, ‐B, ‐DR, and ‐DQ loci [1, 3].

In contrast, antibodies targeting HLA‐C and HLA‐DP loci have long been considered of secondary importance and were therefore frequently excluded from allocation algorithms. This perception was partly driven by the relatively lower and variable surface expression of HLA‐C molecules [3, 4], including on endothelial cells, as well as by uncertainties regarding the clinical relevance of anti‐HLA‐DP antibodies in early transplant cohorts. In addition, historical reliance on serological typing methods and the absence of systematic high‐resolution HLA‐C and HLA‐DP typing in early transplant programmes limited the ability to accurately detect these incompatibilities and to evaluate their clinical impact.

With the widespread implementation of solid‐phase immunoassays and molecular high‐resolution HLA typing [5, 6, 7], an increasing body of evidence has demonstrated the pathogenic potential of anti‐HLA‐C and anti‐HLA‐DP antibodies, particularly their association with antibody‐mediated rejection and impaired graft survival [8, 9, 10, 11, 12, 13]. Despite these advances, these antibodies remain inconsistently integrated into allocation systems [1, 14, 15], creating a potential mismatch between formal allocation rules and clinical decision‐making during donor offer evaluation. In France, the national allocation system managed by the Agence de la biomédecine does not currently include these antibodies in the definition of unacceptable antigens [1]. This gap has resulted in heterogeneous clinical practices across transplant centres, where anti‐HLA‐C and/or anti‐HLA‐DP antibodies are often considered during donor offer evaluation, while allocation scores do not formally reflect this additional immunological constraint.

Such a mismatch between allocation metrics and real‐world clinical decision‐making may lead to an underestimation of immunological risk and contribute to inequities in access to transplantation. Patients whose sensitisation profile includes anti‐HLA‐C and/or anti‐HLA‐DP antibodies may present acceptable cPRA values yet experience prolonged waiting times due to repeated graft refusals at the centre level that are not captured by current allocation metrics. To better characterise the non‐linear consequences of sensitisation on theoretical graft accessibility, refined metrics such as the relative loss of graft access (RLGA) have recently been proposed as complementary tools to classical cPRA estimation [16].

In this context, the present study aimed to comprehensively evaluate the impact of excluding anti‐HLA‐C and anti‐HLA‐DP antibodies from the allocation system, while they are considered in routine clinical decision‐making, on access to kidney transplantation. To address this question, we combined theoretical allocation metrics, donor offer refusal analyses, and observed waiting‐list outcomes in a large French cohort.

## 2 Materials and Methods

### 2.1 Study Design and Population

**FIGURE 1:**
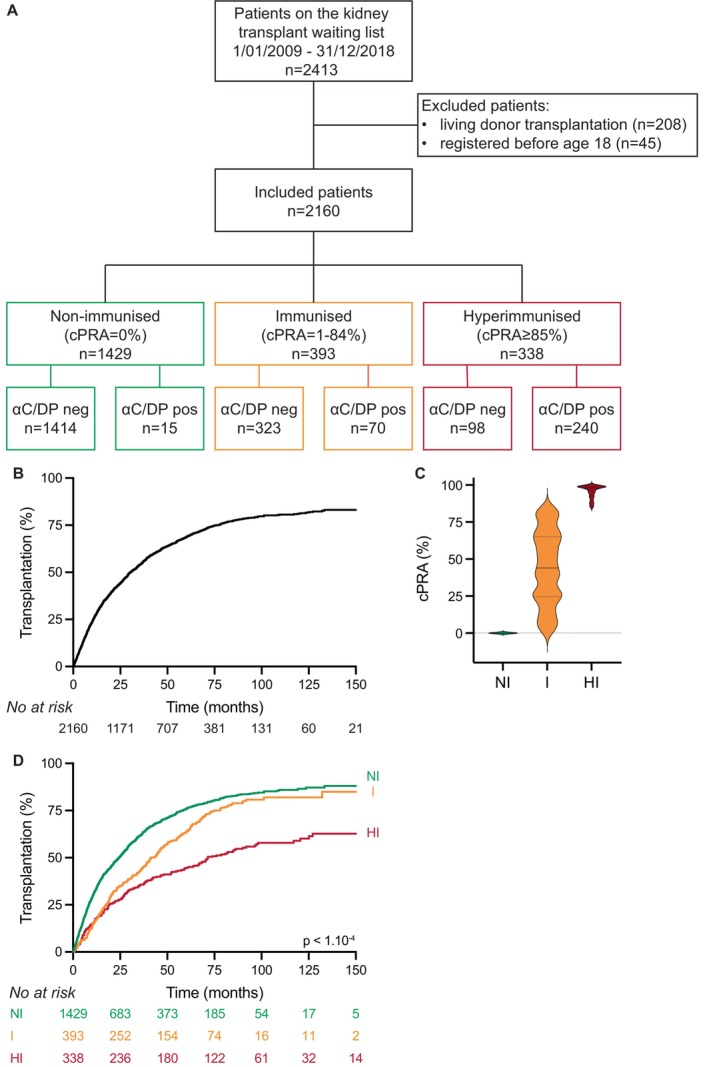
Baseline characteristics of the study population. (A) Flow chart of the study population. Among 2413 patients registered on the Lyon kidney transplant waiting list between January 1, 2009 and December 31, 2018, 2160 were included after exclusion of patients transplanted with a living donor (*n* = 208) and patients registered before 18 years of age (*n* = 45). Included patients were classified according to immunisation status at HLA‐A/B/DR/DQ loci based on cPRA: non‐immunised (NI, cPRA = 0%, *n* = 1429), immunised (I, cPRA 1%–84%, *n* = 393), and hyperimmunised (HI, cPRA ≥ 85%, *n* = 338). For each group, the number of patients with (αC/DP pos) or without (αC/DP neg) anti‐HLA‐C and/or anti‐HLA‐DP antibodies is indicated. (B) Cumulative incidence of kidney transplantation in the overall cohort (*n* = 2160), estimated using the Kaplan–Meier method. (C) Distribution of cPRA values according to immunisation category (NI, I, HI). (D) Cumulative incidence of kidney transplantation stratified by immunisation category (NI, I, HI), estimated using the Kaplan–Meier method. Differences between groups were assessed using the log‐rank test.

This retrospective cohort study included adult patients registered on the kidney transplant waiting list in Lyon (France) between January 1, 2009 and December 31, 2018. Patient data were extracted from the national transplant registry (CRISTAL, Agence de la biomédecine). Exclusion criteria comprised age under 18 years at listing (*n* = 45) and transplantation with a living donor (*n* = 208). Patients listed for another organ at the same time were not included in the extraction. After exclusions, the final study population consisted of 2160 patients (Figure 1A).

Clinical data were obtained from national registry (Cristal; http://www.sipg.sante.fr/portail/). The characteristics of the patients are summarised in Table 1.

**TABLE 1:** Baseline characteristics of the cohort.

Variable	All	No αC/DP	αC/DP	*p*‐value
	(*n* = 2160)	(*n* = 1835)	(*n* = 325)	
Age at listing (years)	51.5 (±14)	51.7 (±14)	50.4 (±13)	0.1
Male, *n* (%)	1335 (61.8%)	1174 (64%)	161 (49.5%)	1 × 10^−6^
ABO blood group, *n* (%)				0.2
O	930 (43.1%)	792 (43.2%)	138 (42.5)	
A	916 (42.4%)	774 (42.2%)	142 (43.7)	
B	230 (10.7%)	203 (11.1%)	21 (8.3%)	
AB	84 (3.9%)	66 (3.6%)	18 (5.5%)	
On dialysis at listing, *n* (%)	1401 (65.5%)	1155 (63.5%)	246 (77.1%)	3 × 10^−6^
Initial nephropathy, *n* (%)				6 × 10^−9^
Glomerular	1004 (46.5%)	878 (47.8%)	126 (38.8%)	
Tubulo‐interstitial	191 (8.8%)	153 (8.3%)	38 (11.7%)	
Vascular	163 (7.6%)	151 (8.2%)	12 (3.7%)	
Polycystic	247 (11.4%)	217 (11.8%)	30 (9.2%)	
Uropathy	44 (2%)	26 (1.4%)	18 (5.5%)	
Other	273 (12.6%)	218 (11.9%)	55 (16.9%)	
Undetermined	238 (11%)	192 (10.5%)	46 (14.2%)	
Rank of transplant, *n* (%)				1 × 10^−98^
1	1782 (82.5%)	1661 (90.5%)	121 (37.2%)	
2	303 (14%)	157 (8.6%)	146 (44.9%)	
3	64 (3%)	16 (0.9%)	48 (14.8%)	
4	10 (0.5%)	0 (0%)	10 (3.1%)	
5	1 (0.1%)	1 (0.1%)	0 (0%)	
Other organ transplant, *n* (%)				0.5
0	2052 (95%)	1744 (95%)	308 (94.8%)	
1	101 (4.7%)	86 (4.7%)	15 (4.6%)	
2	7 (0.3%)	5 (0.3%)	2 (0.6%)	
cPRA, %	22.6% (±36.9)	12% (±26.6)	83% (±28)	7 × 10^−157^

Abbreviation: cPRA, calculated panel reactive antibody.

### 2.2 HLA Antibody Detection and Classification

Anti‐HLA antibodies were detected using HLA class I and class II single‐antigen bead assays (LIFECODES single antigen, Immucor, Werfen, Barcelona, Spain) according to manufacturers' recommendations. For all HLA loci, including HLA‐C and HLA‐DP loci, positivity was defined by a mean fluorescence intensity (MFI) > 500. Antibodies were analysed at the antigen level for allocation metrics. To classify antigens as unacceptable, recommendations from the Société Francophone d'Histocompatibilité et d'Immunogénétique were followed. Immunisation status was defined based on antibodies directed against HLA‐A, ‐B, ‐DR, and ‐DQ loci.

### 2.3 Virtual Crossmatch Practice and Donor Offer Evaluation

In routine clinical practice at our centre, donor offers were systematically evaluated using virtual crossmatch assessment. Although anti‐HLA‐C and anti‐HLA‐DP antibodies were not included in the French national allocation algorithm during the study period, these antibodies were considered during local donor offer evaluation similarly to antibodies directed against classical HLA loci when judged clinically relevant by the transplant team. Virtual crossmatch assessment was performed upstream of organ procurement as part of the routine allocation process.

### 2.4 Calculation of cPRA and RLGA

Classical cPRA values were extrapolated from the national transplant register algorithm (TGI, Cristal, Agence de la biomédecine) considering antibodies against HLA‐A, ‐B, ‐DR, and ‐DQ loci only. A ‘real cPRA’ was then computed by including anti‐HLA‐C and anti‐HLA‐DP antibodies when they were present using the Organ Procurement and Transplantation Network (OPTN) cPRA calculator. Concordance analyses performed in a representative subset of patients demonstrated a strong correlation between the French TGI‐based cPRA calculation and the OPTN cPRA calculator.

The RLGA score was calculated as previously described by Usureau et al. [16], and represents the percentage reduction in residual graft access associated with changes in cPRA between two calculation methods (here, with or without inclusion of anti–HLA‐C/DP antibodies), relative to the maximal cPRA value (100%). This metric accounts for the non‐linear relationship between cPRA and transplant accessibility, whereby identical numerical increases in cPRA result in greater relative losses of graft access at higher cPRA levels. Patients with anti‐C/DP antibodies and a baseline cPRA of 100% (*n* = 86) were excluded from RLGA analyses, as their theoretical access was already null.

### 2.5 Statistical Analysis

Continuous variables were expressed as means ± standard deviations (SD), whereas categorical variables were presented as counts and percentages. Comparisons between independent groups were performed using Student's *t*‐test or the Mann–Whitney *U* test for continuous variables and chi‐square or Fisher's exact tests for categorical variables, as appropriate.

For paired comparisons of classical cPRA and recalculated cPRA values after inclusion of anti‐HLA‐C and/or anti‐HLA‐DP antibodies, the Wilcoxon signed‐rank test was used. Differences in cPRA change between antibody‐specificity groups were assessed using the Kruskal–Wallis test followed by pairwise Wilcoxon rank‐sum tests with false discovery rate correction. Comparisons of RLGA across groups were also performed using the Kruskal–Wallis test.

Time to transplantation was analysed using Kaplan–Meier methods, and differences between groups were assessed using the log‐rank test. Cox proportional hazards regression models were used to estimate hazard ratios (HR) and 95% confidence intervals (CI) for access to transplantation in univariable and multivariable analyses, with transplantation as the event of interest. Covariates included age, sex, ABO blood group, cPRA category, dialysis status at listing, initial nephropathy, retransplantation, and previous transplantation with another organ. Variables associated with transplantation in univariable analyses (*p* < 0.05) were entered into multivariable models.

All statistical analyses were performed using R version 4.4.3 (R Foundation for Statistical Computing, Vienna, Austria). A two‐sided *p* value < 0.05 was considered statistically significant.

### 2.6 Artificial Intelligence Use Statement

Artificial intelligence tools were used solely for language editing and manuscript preparation. No artificial intelligence tool was used for data analysis, interpretation of results, or generation of scientific content. All authors reviewed and approved the final manuscript and take full responsibility for its content.

### 2.7 Ethics Statement

The study was conducted in accordance with the Declaration of Helsinki and French legislation governing biomedical research. This retrospective observational study involved secondary analysis of routinely collected clinical and registry data without any additional intervention or modification of patient management. It was classified as non‐interventional research under the French Jardé law (RIPH category 3) and complied with national data protection requirements and the General Data Protection Regulation (GDPR, MR‐004).

## 3 Results

### 3.1 Baseline Characteristics of the Study Population

The study cohort comprised 2160 adult patients registered on the kidney transplant waiting list in Lyon between January 1, 2009 and December 31, 2018 (Figure 1A). Among these patients, 61.8% were men, with a mean age of 51.5 years at listing (Table 1). Most patients were on dialysis at the time of registration on the waiting list (65.5%; Table 1). The median waiting time to kidney transplantation was 30.3 months (95% CI, 28.6–33.3; Figure 1B), consistent with national waiting time estimates [17].

Based on antibodies directed against HLA‐A, ‐B, ‐DR, and ‐DQ loci, 1429 patients (66.2%) were classified as non‐immunised (NI, cPRA = 0%), 393 patients (18.2%) as immunised (I, cPRA = [1%–84%]), and 338 patients (15.6%) as hyperimmunised (HI, cPRA ≥ 85%), according to national criteria (Figure 1C). Median waiting time differed across immunisation categories, with immunised and hyperimmunised patients experiencing reduced access to transplantation compared with non‐immunised patients (respectively 41.4 months for I and 71.5 months for HI vs. 24.4 months for NI, *p* < 1 × 10^−4^; Figure 1D).

### 3.2 Prevalence and Distribution of Anti‐HLA‐C and ‐DP Antibodies

**FIGURE 2:**
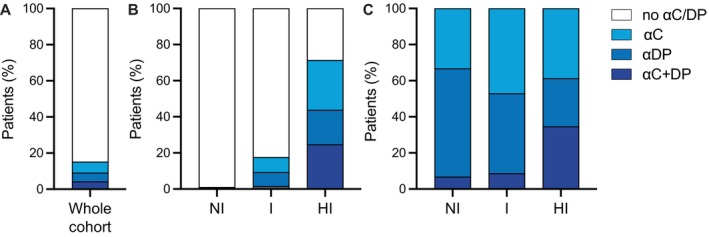
Prevalence and distribution of anti‐HLA‐C and ‐DP antibodies. (A) Distribution of anti‐HLA‐C only, anti‐HLA‐DP only, and combined anti‐HLA‐C/DP antibodies in the whole cohort (*n* = 2160). Percentages are expressed relative to the entire study population. (B) Prevalence and antibody profile of anti‐HLA‐C and/or anti‐HLA‐DP antibodies according to immunisation status at classical HLA loci (non‐immunised [NI], immunised [I], and hyperimmunised [HI]). Percentages are expressed relative to each immunisation group. (C) Distribution of anti‐HLA‐C only, anti‐HLA‐DP only, and combined anti‐HLA‐C/DP antibody profiles among patients positive for anti‐HLA‐C and/or anti‐HLA‐DP antibodies within each immunisation group. Percentages are expressed relative to anti‐HLA‐C/DP–positive patients.

Anti‐HLA‐C and/or anti‐HLA‐DP antibodies were detected in 325 patients, representing 15.1% of the overall cohort (Figure 2A). Among these patients, 131 (40.3%) had anti‐HLA‐C antibodies alone, 104 (32.0%) had anti‐HLA‐DP antibodies alone, and 90 (27.7%) had both anti‐HLA‐C and anti‐HLA‐DP antibodies (Figure 2A).

The prevalence of anti‐HLA‐C and/or anti‐HLA‐DP antibodies increased markedly across immunisation categories. While these antibodies were rare among non‐immunised patients (1.1%), they were detected in 17.8% of immunised patients and in 71% of hyperimmunised patients, indicating a strong association between anti‐HLA‐C/DP antibodies and global sensitisation (Figure 2B). Within the non‐immunised and immunised groups, most patients presented isolated anti‐HLA‐C antibodies (33.3% and 47.1%, respectively) or isolated anti‐HLA‐DP antibodies (60.0% and 44.3%, respectively; Figure 2C). In contrast, among hyperimmunised patients, anti‐HLA‐C antibodies were detected in 38.7%, anti‐HLA‐DP antibodies in 26.7%, and the proportion of patients with combined anti‐HLA‐C and anti‐HLA‐DP antibodies was markedly increased to 34.6% (Figure 2C).

### 3.3 Impact of Anti‐HLA‐C/DP Antibodies on cPRA

We first assessed the impact of anti‐HLA‐C and anti‐HLA‐DP antibodies on cPRA. For this purpose, cPRA was recalculated for all patients with anti‐HLA‐C and/or anti‐HLA‐DP antibodies by incorporating these specificities into the list of unacceptable antigens, yielding a ‘real cPRA’.

**FIGURE 3:**
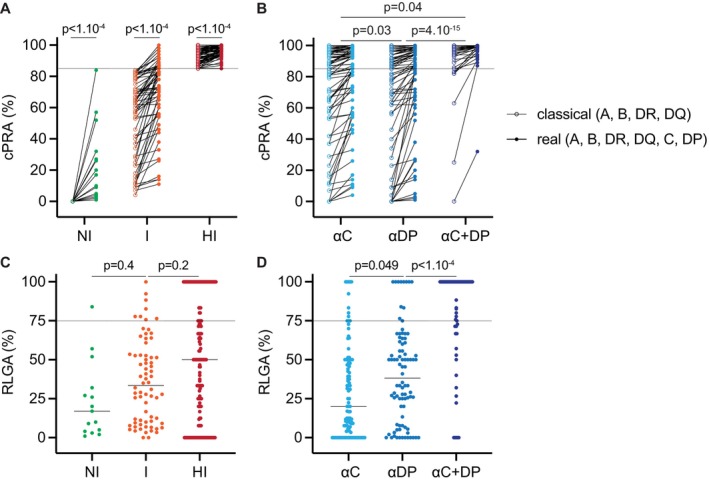
Impact of anti‐HLA‐C/DP antibodies on theoretical access to kidney transplantation. (A) Calculated panel reactive antibody (cPRA) values according to immunisation status at classical HLA loci (non‐immunised [NI], immunised [I], and hyperimmunised [HI]) for patients with anti‐HLA‐C/DP antibodies (*n* = 325). For each patient, cPRA calculated using classical loci only (HLA‐A, ‐B, ‐DR, ‐DQ) is compared with cPRA recalculated after inclusion of anti‐HLA‐C and/or anti‐HLA‐DP antibodies (real cPRA; HLA‐A, ‐B, ‐DR, ‐DQ, ‐C, ‐DP). Paired comparisons were performed using the Wilcoxon signed‐rank test. (B) Change in cPRA according to antibody specificity among patients with anti‐HLA‐C only (αC) (*n* = 131), anti‐HLA‐DP only (αDP) (*n* = 104), or combined anti‐HLA‐C and anti‐HLA‐DP antibodies (αC + DP) (*n* = 90). For each patient, classical cPRA and real cPRA values are shown. Within each group, paired comparisons were assessed using the Wilcoxon signed‐rank test. Differences in change between groups were evaluated using the Kruskal–Wallis test followed by Wilcoxon rank‐sum tests with false discovery rate correction. (C) Relative loss of graft access (RLGA) according to immunisation status (NI, I, HI) among patients with anti‐HLA‐C and/or anti‐HLA‐DP antibodies and a classical cPRA < 100% (*n* = 239). Horizontal bars indicate median values. Differences between groups were assessed using the Kruskal–Wallis test. (D) RLGA according to antibody specificity (anti‐HLA‐C only, anti‐HLA‐DP only, and combined anti‐HLA‐C/DP antibodies). Horizontal bars indicate median values. Group comparisons were performed using the Kruskal–Wallis test.

Including anti‐HLA‐C and/or anti‐HLA‐DP antibodies resulted in a near‐systematic increase in cPRA values across all immunisation groups (mean increase of 23.3 ± 24.3 in non‐immunised patients, 15.8 ± 15.0 in immunised patients, and 1.3 ± 2.1 in hyperimmunised patients; Figure 3A). Among the 85 patients with anti‐HLA‐C and/or anti‐HLA‐DP antibodies within the non‐immunised and immunised groups, 22 patients (25.9%) reached a cPRA ≥ 85% (Figure 3A), corresponding to the threshold defining hyperimmunised patients, who would therefore have been eligible for higher allocation priority according to the French allocation programme.

The increase in cPRA was greater in patients with anti‐HLA‐DP antibodies than in those with anti‐HLA‐C antibodies (mean increase: 8.0 ± 14.4 vs. 5.1 ± 9.9, *p* = 0.03; Figure 3B). In contrast, the combined presence of anti‐HLA‐C and anti‐HLA‐DP antibodies had a more limited incremental impact on cPRA compared with isolated anti‐HLA‐C or anti‐HLA‐DP antibodies (mean increase: 3.0 ± 8.2 vs. 5.1 ± 9.9, *p* = 0.04; and 3.0 ± 8.2 vs. 8.0 ± 14.4, *p* = 4 × 10^−5^, respectively; Figure 3B), largely because 92.2% of these patients already belonged to the hyperimmunised group.

### 3.4 Non‐Linear Impact of Anti‐HLA‐C/DP Antibodies on Theoretical Graft Accessibility

Although increases in cPRA after inclusion of additional unacceptable antigens are mathematically expected, identical numerical changes in cPRA do not uniformly reflect the actual loss of access to transplantation across patients with different baseline sensitisation levels. An identical absolute increase in cPRA may have minimal consequences in patients with low levels of sensitisation but represents a substantial reduction in compatible donor availability in highly sensitised patients. In this context, the RLGA was used as a complementary metric to better illustrate the non‐linear impact of additional unacceptable antigens on theoretical graft accessibility. We therefore calculated the RLGA in the 239 patients immunised against HLA‐C and/or HLA‐DP and with a classical cPRA < 100%. Among these patients, 83.5% experienced a reduction in theoretical access to transplantation when these antibodies were taken into account. Notably, 21.5% of patients lost more than 75% of their theoretical access, a threshold associated with a significant increase in observed waiting time in our cohort (*p* < 1 × 10^−4^, Figures 3C and S1A). Among these patients, 76.7% even lost all theoretical access to transplantation, as reflected by RLGA values approaching 100%.

RLGA values increased from non‐immunised to immunised and hyperimmunised groups, but substantial interindividual variability was observed within each category. Importantly, several patients with moderate classical cPRA values exhibited very high RLGA scores once anti‐HLA‐C and/or anti‐HLA‐DP antibodies were included (Figure 3C,D), highlighting the limitations of cPRA alone in capturing the non‐linear consequences of sensitisation on transplant accessibility.

In conclusion, inclusion of anti‐HLA‐C/DP antibodies significantly impacts theoretical allocation metrics and may substantially reduce estimated graft accessibility despite apparently moderate classical cPRA values.

### 3.5 Observed Access to Transplantation

**FIGURE 4:**
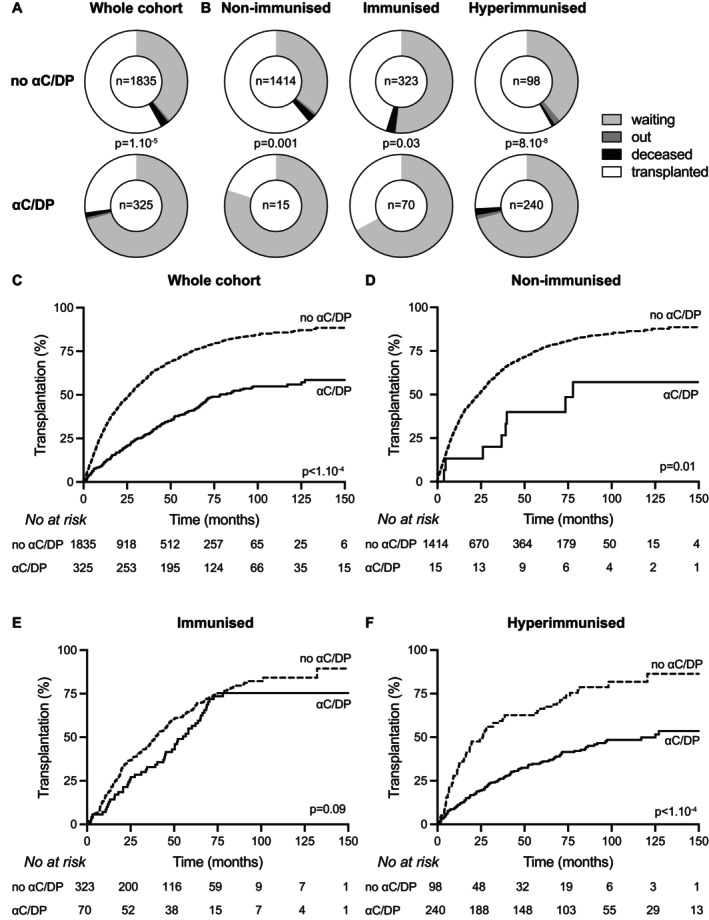
Impact of anti‐HLA‐C/DP antibodies on observed access to kidney transplantation. (A, B) Status at 3 years after listing in the whole cohort (A) and according to immunisation status at classical HLA loci (non‐immunised, immunised, and hyperimmunised) (B), stratified by the presence (αC/DP) or absence (no αC/DP) of anti‐HLA‐C and/or anti‐HLA‐DP antibodies. Pie charts represent the proportion of patients who were transplanted, still on waiting list, or removed from the waiting list due to death or other reasons. Comparisons between patients with and without anti‐HLA‐C/DP antibodies within the whole cohort or each immunisation category were performed using the chi‐square test. (C–F) Cumulative incidence of kidney transplantation over time according to the presence (αC/DP) or absence (no αC/DP) of anti‐HLA‐C and/or anti‐HLA‐DP antibodies in the whole cohort (C), and in non‐immunised (D), immunised (E), and hyperimmunised patients (F). Curves were estimated using the Kaplan–Meier method, and differences between groups were assessed using the log‐rank test.

Building on these results, we assessed the impact of anti‐HLA‐C and/or anti‐HLA‐DP antibodies on access to kidney transplantation at 3 years after listing, which corresponds approximately to the median waiting time in our cohort. We therefore focused on patient outcomes at this time point, anticipating that less than 50% of patients would remain on the waiting list. We observed that the proportion of patients still awaiting transplantation at 3 years was significantly higher among those with anti–HLA‐C/DP antibodies compared with patients without these antibodies (70.5% vs. 38.7%, *p* = 1 × 10^−5^; Figure 4A). This difference was observed across all immunisation categories but was particularly pronounced in non‐immunised and hyperimmunised patients (Figure 4B). Among non‐immunised patients, the proportion of patients still waiting at 3 years was 80.0% in patients with anti‐HLA‐C/DP antibodies versus 35.7% in those without anti‐HLA‐C/DP antibodies (*p* = 0.001; Figure 4B). Similarly, among hyperimmunised patients, 70.8% of patients with anti‐HLA‐C/DP antibodies remained on the waiting list at 3 years, compared with only 38.8% of patients without anti‐HLA‐C/DP antibodies (*p* = 8 × 10^−8^; Figure 4B). A significant, albeit less pronounced, effect was also observed among immunised patients (67.1% vs. 51.7%, *p* = 0.03; Figure 4B). Beyond this static analysis, we evaluated the impact of anti‐HLA‐C and/or anti‐HLA‐DP antibodies on the cumulative incidence of transplantation over time. In the overall cohort, the presence of anti‐HLA‐C/DP antibodies was associated with a significantly reduced access to transplantation (*p* < 1 × 10^−4^) (Figures 4C and S2A). The most reduced access was observed in patients harbouring both anti‐HLA‐C and anti‐HLA‐DP antibodies (Figure S2A). Because patients with anti‐HLA‐C/DP antibodies were predominantly immunised or hyperimmunised and therefore had higher baseline cPRA values, we further analysed access to transplantation according to immunisation status. The deleterious effect of anti‐HLA‐C/DP antibodies on access to transplantation persisted in non‐immunised patients (*p* = 0.01) and in hyperimmunised patients (*p* < 1 × 10^−4^) and showed a strong trend towards significance in immunised patients (*p* = 0.09; Figure 4D–F).

### 3.6 Multivariable Analysis of Access to Transplantation

Although univariable analyses showed that the presence of anti‐HLA‐C and/or anti‐HLA‐DP antibodies was associated with reduced access to kidney transplantation, this association could be influenced by multiple confounding factors. We therefore compared baseline characteristics of patients with and without anti‐HLA‐C/DP antibodies (Table 1). Patients with anti‐HLA‐C/DP antibodies differed significantly from those without these antibodies in several aspects. They presented different types of initial nephropathies, were more often on dialysis at listing (77.1% vs. 63.5%, *p* = 3 × 10^−6^), and were more often retransplanted (62.8% vs. 9.6%, *p* = 1 × 10^−98^). As expected, they also displayed markedly higher levels of sensitisation, with a mean cPRA of 83% compared with 12% in patients without anti‐HLA‐C/DP antibodies (*p* = 7 × 10^−157^). In contrast, the proportion of men was lower in the anti‐HLA‐C/DP group (49.5% vs. 64.0%, *p* = 1 × 10^−6^).

To determine whether anti‐HLA‐C and/or anti‐HLA‐DP antibodies were independently associated with reduced access to transplantation, we performed a multivariable analysis using a Cox proportional hazards regression model. In univariable analyses, several variables were significantly associated with reduced access to transplantation, including anti‐HLA‐C/DP immunisation, age, ABO blood group, dialysis status, initial nephropathy, history of previous transplantation, and HLA‐A/B/DR/DQ sensitisation (Table 2, left column).

**TABLE 2:** Univariable and multivariable analyses of factors associated with access to kidney transplantation.

Variable	No. of patients	Univariable			Multivariable		
		HR	95% CI	*p*‐value	HR	95% CI	*p*‐value
Age							
[40–65]	1278	1.00	Reference				
< 40	500	1.48	[1.32–1.66]	2 × 10^−11^	**1.58**	**[1.41–1.79]**	**5** **× 10** ^**−14**^
> 65	382	0.97	[0.84–1.11]	0.63	0.92	[0.80–1.07]	0.28
Sex							
Male	1335	1.00	Reference				
Female	825	1.01	[0.92–1.12]	0.78	NA	NA	NA
ABO blood group							
A	916	1.00	Reference				
O	930	0.64	[0.58–0.72]	3 × 10^−16^	**0.60**	**[0.54–0.67]**	**3 × 10** ^**−20**^
B	230	0.69	[0.59–0.82]	2 × 10^−5^	**0.61**	**[0.51–0.72]**	**2 × 10** ^**−8**^
AB	84	1.00	[0.77–1.29]	0.97	1.08	[0.84–1.40]	0.54
On dialysis at listing							
No	737	1.00	Reference				
Yes	1401	1.12	[1.01–1.25]	0.03	**1.30**	**[1.17–1.45]**	**1 × 10** ^**−6**^
Initial nephropathy							
Glomerular	1004	1.00	Reference				
Tubulo‐interstitial	191	0.72	[0.60–0.87]	5 × 10^−4^	**0.82**	**[0.67–0.99]**	**0.04**
Vascular	163	0.92	[0.76–1.11]	0.37	0.93	[0.77–1.13]	0.47
Polycystic	247	0.94	[0.80–1.10]	0.45	1.07	[0.91–1.26]	0.38
Uropathy	44	0.78	[0.55–1.11]	0.17	1.00	[0.70–1.43]	1.00
Other	273	0.85	[0.72–0.99]	0.04	0.94	[0.80–1.11]	0.47
Undetermined	238	0.96	[0.82–1.13]	0.62	0.97	[0.82–1.15]	0.70
Rank of transplant							
Per additional transplant	2160	0.56	[0.50–0.64]	4 × 10^−21^	**0.76**	**[0.66–0.87]**	**1 × 10** ^**−4**^
Other organ transplant							
Per additional transplant	2160	0.67	[0.53–0.85]	9 × 10^−4^	**0.76**	**[0.59–0.98]**	**0.03**
HLA‐A/B/DR/DQ sensitisation							
cPRA = 0	1445	1.00	Reference				
cPRA = [1–84]	377	0.73	[0.64–0.83]	2 × 10^−6^	**0.83**	**[0.72–0.95]**	**0.006**
cPRA ≥ 85	338	0.44	[0.38–0.52]	1 × 10^−24^	**0.75**	**[0.60–0.94]**	**0.01**
HLA‐C/DP sensitisation							
No anti‐HLA‐C/DP	1835	1.00	Reference				
Anti‐HLA‐C/DP	325	0.40	[0.34–0.47]	2 × 10^−28^	**0.52**	**[0.42–0.66]**	**2 × 10** ^**−8**^

*Note:* Variables with a *p*‐value < 0.05 in the univariable model were incorporated into the multivariable model. Bold values indicate variables that remained statistically significant in the multivariable analysis.

Abbreviations: 95% CI, confidence interval; HR, hazard ratio; NA, not applicable.

**FIGURE 5:**
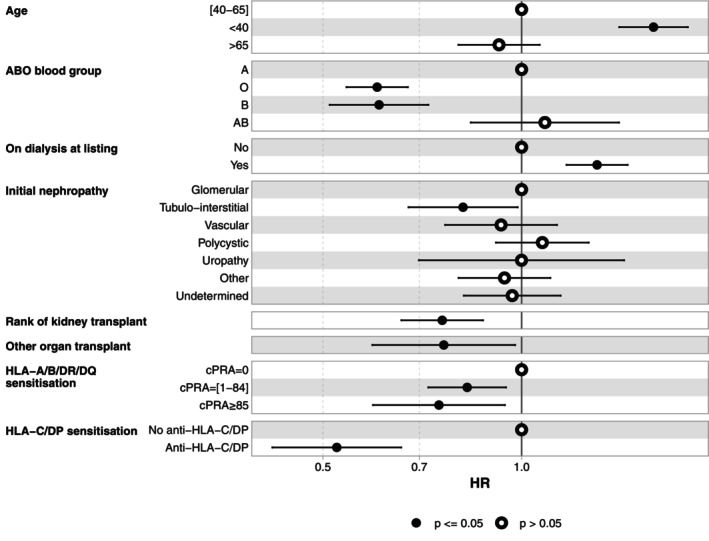
Multivariable analyses of factors associated with access to kidney transplantation. Hazard ratios (HR) and 95% confidence intervals (CI) were obtained from a multivariable Cox proportional hazards model, including only variables with *p* < 0.05 in univariable analyses. A HR < 1 indicates a lower likelihood of receiving a transplant.

When these variables were included in the multivariable model (Table 2, right column), anti‐HLA‐C/DP immunisation remained independently associated with reduced access to kidney transplantation (hazard ratio [HR] = 0.52; 95% CI, 0.42–0.66; *p* = 2 × 10^−8^; Table 2, right column; Figure 5). This was also true if they were considered separately (Figure S3A). In addition, blood groups O and B (HR = 0.60; 95% CI, 0.54–0.67; *p* = 3 × 10^−20^ and HR = 0.61; 95% CI,0.51–0.72; *p* = 2 × 10^−8^, respectively), kidney retransplantation (HR = 0.76; 95% CI, 0.66–0.87; *p* = 1 × 10^−4^), previous transplantation of another organ (HR = 0.76; 95% CI, 0.59–0.98; *p* = 0.03), anti‐HLA‐A/B/DR/DQ immunisation (cPRA 1%–84%) (HR = 0.83; 95% CI, 0.72–0.95; *p* = 0.006) and hyperimmunisation (cPRA ≥ 85%) (HR = 0.75; 95% CI, 0.60–0.94; *p* = 0.01) were also independently associated with a lower probability of transplantation (Table 2, right column; Figure 5). Conversely, patients aged < 40 (HR = 1.58; 95% CI, 1.41–1.79; *p* = 5 × 10^−14^) and patients already on dialysis at listing had a higher probability of transplantation (HR = 1.30; 95% CI, 1.17–1.45; *p* = 1 × 10^−6^) (Table 2, right column; Figure 5).

Taken together, these results indicate that the presence of anti‐HLA‐C and/or anti‐HLA‐DP antibodies is independently associated with reduced access to kidney transplantation, even after adjustment for major demographic, clinical, and immunological confounders.

### 3.7 Association of Anti‐HLA‐C/DP Antibodies With Immunological Donor Offer Refusals

To further explore the mechanisms underlying the reduced transplant accessibility associated with anti‐HLA‐C/DP antibodies, we analysed donor offer refusal patterns in our cohort. A total of 8403 donor offers were recorded during the study period, among which 1154 lacked identifiable recipient information and could not be linked to individual patients. After matching donor offers with waiting‐list data, analyses included 1861 patients from the study cohort.

**FIGURE 6:**
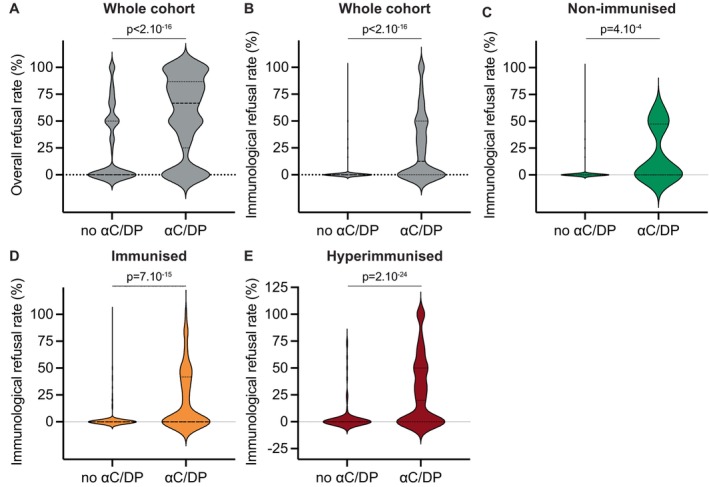
Donor offer refusal rates according to anti‐HLA‐C/DP antibody status. (A) Overall donor offer refusal rates in patients without (no αC/DP) or with anti‐HLA‐C and/or anti‐HLA‐DP antibodies (αC/DP) in the whole cohort. (B–E) Immunological donor offer refusal rates according to anti‐HLA‐C/DP antibody status in the whole cohort (B), non‐immunised patients (C), immunised patients (D), and hyperimmunised patients (E). Violin plots represent the distribution of refusal rates in each group. Dashed horizontal lines indicate quartiles and median values. Comparisons between groups were performed using the Wilcoxon rank‐sum test.

Patients harbouring anti‐HLA‐C and/or anti‐HLA‐DP antibodies exhibited significantly higher overall donor offer refusal rates compared with patients without these antibodies (mean 56% vs. 27.6%, *p* < 2 × 10^−16^, Figure 6A). More importantly, immunological refusal rates were markedly increased in patients with anti‐HLA‐C/DP antibodies (mean 25.7% vs. 1.8%, *p* < 2 × 10^−16^, Figure 6B), including within non‐immunised (mean 18.6% vs. 1.4%, *p* = 4 × 10^−4^, Figure 6C), immunised (mean 20.1% vs. 2.6%, *p* = 7 × 10^−15^, Figure 6D), and hyperimmunised groups (mean 27.9% vs. 4.5%, *p* = 2 × 10^−24^, Figure 6E). This effect was observed for isolated anti‐HLA‐C antibodies, isolated anti‐HLA‐DP antibodies, and was most pronounced in patients harbouring both anti‐HLA‐C and anti‐HLA‐DP antibodies (*p* < 2 × 10^−16^, Figure S4A).

These findings support the hypothesis that donor offers formally considered compatible according to the national allocation algorithm were subsequently declined following local virtual crossmatch assessment incorporating anti‐HLA‐C/DP antibodies.

## 4 Discussion

In this study, we comprehensively evaluated the impact of excluding anti‐HLA‐C and anti‐HLA‐DP antibodies from allocation algorithms, despite their routine consideration by transplant clinicians, on access to kidney transplantation in a large French cohort. By combining theoretical allocation metrics with observed waiting list outcomes, we demonstrate that the presence of anti‐HLA‐C and/or anti‐HLA‐DP antibodies is consistently associated with reduced access to transplantation when these antibodies are not incorporated into allocation systems. This effect was observed across multiple complementary indicators, including higher cPRA values after antibody inclusion, reduced theoretical access as estimated by the RLGA, a greater proportion of patients remaining on the waiting list beyond 3 years, a lower cumulative incidence of transplantation, and an independent association with reduced transplantation probability in multivariable analysis.

Historically, the limited consideration of anti‐HLA‐C and anti‐HLA‐DP antibodies in allocation systems may be explained by the lower surface expression of HLA‐C molecules [3, 4] and by the late implementation of systematic HLA‐C and DP typing. However, clinical decision‐making has evolved more rapidly than allocation algorithms. In daily practice, many transplant centres now take anti‐HLA‐C and anti‐HLA‐DP antibodies into account when evaluating donor offers, even when these antibodies are not formally included in allocation scores. Our results highlight the consequences of this discrepancy, showing that patients harbouring these antibodies may experience reduced access to transplantation that is not adequately captured by current allocation metrics. Moreover, underestimation of their level of sensitisation may prevent these patients from benefiting from specific programmes dedicated to highly sensitised candidates, such as, in France, the ‘Hyperimmunised with Permissible Antigens’ programme for patients with a cPRA ≥ 85%, or access to desensitisation therapies such as imlifidase for those with a cPRA ≥ 98% [18].

Inclusion of anti‐HLA‐C and anti‐HLA‐DP antibodies into allocation algorithms could potentially increase the number of candidates classified as hyperimmunised and therefore eligible for priority allocation programmes. However, in our cohort, most patients harbouring anti‐HLA‐C/DP antibodies already belonged to the hyperimmunised group according to current French allocation criteria. Consequently, the overall impact on the number of highly prioritised candidates would likely remain limited. Rather than massively increasing prioritisation volumes, integration of these antibodies may primarily improve identification of patients whose real‐world transplant accessibility is underestimated by current allocation metrics. More broadly, these findings raise the question of whether cPRA prioritisation thresholds themselves may need to evolve alongside broader integration of immunological constraints into allocation frameworks, as recently suggested by Bertrand et al. in analyses of transplant accessibility according to sensitisation levels [19].

Beyond transplant accessibility alone, integration of anti‐HLA‐C and anti‐HLA‐DP antibodies into allocation algorithms may also have implications for long‐term transplant outcomes. The rationale for considering these antibodies during donor offer evaluation is based on accumulating evidence linking donor‐specific anti‐HLA‐C and anti‐HLA‐DP antibodies with antibody‐mediated rejection and impaired graft survival [8, 9, 10, 11, 12, 13]. Improved integration of these antibodies into allocation frameworks could therefore not only enhance consistency between allocation rules and clinical practice, but also potentially reduce transplantation across clinically relevant immunological incompatibilities and contribute to improved long‐term graft success.

Our analyses of donor offer refusal patterns strengthen the mechanistic interpretation of these findings. In our centre, donor offers were routinely evaluated using virtual crossmatch assessment incorporating anti‐HLA‐C and anti‐HLA‐DP antibodies, despite these antibodies not being formally integrated into the national allocation algorithm. Consequently, some donor offers formally considered compatible by the allocation system were declined because of predicted anti‐HLA‐C/DP incompatibilities. Consistent with this mechanism, patients harbouring anti‐HLA‐C/DP antibodies exhibited significantly higher rates of immunological donor offer refusals. Although multiple clinical factors may contribute to donor offer refusal, these observations support the role of virtual crossmatch assessment as a major contributor to the reduced transplant probability observed in our cohort. More broadly, our findings illustrate how clinical immunological risk assessment may evolve more rapidly than allocation frameworks, creating discrepancies between theoretical allocation priority and real‐world transplant accessibility.

However, these discrepancies between allocation algorithms and local clinical practices may also have broader systemic implications for the national allocation network. In the French allocation system, donor offers are generally issued upstream of organ procurement, and virtual crossmatch assessment is performed early during the allocation process. Consequently, refusals related to anti‐HLA‐C/DP incompatibilities are unlikely to substantially prolong allocation chains or significantly increase cold ischemia times. Nevertheless, integrating additional unacceptable antigens into allocation algorithms may redistribute rather than completely eliminate existing inequities in transplant access. These considerations highlight the balance between immunological risk assessment, equity, utility, and logistical efficiency in kidney allocation systems.

The cPRA score remains a cornerstone of kidney allocation systems, but our results illustrate its limitations when used as the sole indicator of immunological constraint [16]. An identical absolute increase in cPRA does not reflect the same loss of access to transplantation across patients with different baseline levels of sensitisation. In this context, anti‐HLA‐C and anti‐HLA‐DP antibodies may have a limited effect on cPRA while substantially reducing the pool of acceptable donors. RLGA was therefore used as a complementary metric to illustrate the non‐linear impact of sensitisation on theoretical graft accessibility, particularly in highly sensitised patients. Although the increase in cPRA observed after inclusion of additional unacceptable antigens is mathematically expected, high RLGA values were associated with prolonged waiting times in our cohort, including in patients with moderate classical cPRA values. Importantly, a RLGA threshold of 75% or higher was associated with a significant increase in observed waiting time. Therefore, the main contribution of our study is not the demonstration that cPRA mathematically increases after inclusion of additional loci, but rather the quantification of the clinical consequences of this discrepancy within a real‐world allocation framework.

Our analyses also suggest differential effects of anti‐HLA‐C and anti‐HLA‐DP antibodies. While anti‐HLA‐DP antibodies have a stronger impact on cPRA, anti‐HLA‐C antibodies are associated with a comparable reduction in access to transplantation despite a more limited effect on calculated incompatibility. These findings may be explained, firstly, by the lower diversity of HLA‐DP antigens, which confers a greater impact of each anti–HLA‐DP antibody on cPRA (the 4 most frequent HLA‐DP alleles represent 92% of all HLA‐DP alleles in the population), and, on the other hand, by the strong linkage disequilibrium existing between HLA‐C and HLA‐B loci, resulting in lower impact on cPRA increase when the patient is already immunised against HLA‐B alleles.

The limited integration of anti‐HLA‐C and anti‐HLA‐DP antibodies into allocation algorithms is not specific to the French system. International kidney allocation programmes display substantial heterogeneity in the definition of unacceptable antigens and in the consideration of extended HLA loci [1, 14, 15, 20, 21, 22]. Consequently, discrepancies between theoretical prioritisation and real‐world access to transplantation may also exist in other settings, suggesting that our findings reflect a broader challenge in transplant immunology.

This study has several strengths, including the size of the cohort, the use of real‐world waiting list data, and the combined analysis of theoretical and observed access to transplantation. Several limitations should also be acknowledged. First, this was a single‐centre study, and allocation practices may vary across transplant centres, potentially limiting generalisability. Moreover, our analyses were conducted within the specific context of the French kidney allocation system, which may limit generalisability to other allocation frameworks. Second, cPRA calculations relied on two different allocation systems (French TGI‐based calculation and OPTN calculator), although concordance analyses demonstrated a strong correlation between both methods. Finally, antibodies directed against other loci, such as HLA‐DRB3/4/5, HLA‐DQA1 and HLA‐DPA1, as well as allele‐specific antibodies were not analysed and may also contribute to immunological risk and access to transplantation.

In conclusion, our study demonstrates that the discrepancy between allocation rules and clinical decision‐making is associated with reduced access to transplantation for patients carrying anti‐HLA‐C and/or anti‐HLA‐DP antibodies. Future multicentre studies are warranted to validate these findings and to determine how integrating these antibodies and refined immunological metrics into allocation frameworks could improve equity and accuracy in transplant prioritisation.

*Supporting Information*: The Supporting Information includes additional figures illustrating the association between relative loss of graft access (RLGA) and transplant accessibility, transplantation access according to anti‐HLA‐C and anti‐HLA‐DP antibody profiles, multivariable analyses stratified by antibody specificity, and immunological donor offer refusal rates according to anti‐HLA‐C and anti‐HLA‐DP antibody profiles.

## Conflicts of Interest

The authors declare no conflicts of interest.

## Supporting information


**Figure S1:** Association between relative loss of graft access and observed access to transplantation.
**Figure S2:** Observed access to transplantation according to anti‐HLA‐C and anti‐HLA‐DP antibody profiles.
**Figure S3:** Immunological donor offer refusal rates according to anti‐HLA‐C and anti‐HLA‐DP antibody profiles.

**Figure S4:** Multivariable analyses of factors associated with access to kidney transplantation according to anti‐HLA‐C and anti‐HLA‐DP antibody profiles.

## Data Availability

The data that support the findings of this study are available from the corresponding author upon reasonable request.

## References

[1] B. Audry , E. Savoye , M. Pastural , et al., “The New French Kidney Allocation System for Donations After Brain Death: Rationale, Implementation, and Evaluation,” American Journal of Transplantation 22, no. 12 (2022): 2855–2868, 10.1111/ajt.17180. 36000787

[2] N. Mamode , O. Bestard , F. Claas , et al., “European Guideline for the Management of Kidney Transplant Patients With HLA Antibodies: By the European Society for Organ Transplantation Working Group,” Transplant International 35 (2022): 10511, 10.3389/ti.2022.10511. 36033645 PMC9399356

[3] J. Visentin , L. Couzi , and J. L. Taupin , “Clinical Relevance of Donor‐Specific Antibodies Directed at HLA‐C: A Long Road to Acceptance,” HLA 97, no. 1 (2021): 3–14, 10.1111/tan.14106. 33052032

[4] B. S. Carey , K. V. Poulton , and A. Poles , “HLA‐C Expression Level in Both Unstimulated and Stimulated Human Umbilical Vein Endothelial Cells Is Defined by Allotype,” HLA 95, no. 6 (2020): 532–542, 10.1111/tan.13852. 32107874

[5] R. Pei , J. H. Lee , N. J. Shih , M. Chen , and P. I. Terasaki , “Single Human Leukocyte Antigen Flow Cytometry Beads for Accurate Identification of Human Leukocyte Antigen Antibody Specificities,” Transplantation 75, no. 1 (2003): 43–49, 10.1097/01.TP.0000040431.80510.98. 12544869

[6] N. El‐Awar , J. Lee , and P. I. Terasaki , “HLA Antibody Identification With Single Antigen Beads Compared to Conventional Methods,” Human Immunology 66, no. 9 (2005): 989–997, 10.1016/j.humimm.2005.07.005. 16360839

[7] C. Rosser and D. Sage , “Approaches for the Characterization of Clinically Relevant Pre‐Transplant Human Leucocyte Antigen (HLA) Antibodies in Solid Organ Transplant Patients,” International Journal of Immunogenetics 48, no. 5 (2021): 385–402, 10.1111/iji.12552. 34346180

[8] O. Aubert , M. C. Bories , C. Suberbielle , et al., “Risk of Antibody‐Mediated Rejection in Kidney Transplant Recipients With Anti‐HLA‐C Donor‐Specific Antibodies,” American Journal of Transplantation 14, no. 6 (2014): 1439–1445, 10.1111/ajt.12709. 24804568

[9] T. Bachelet , C. Martinez , A. Del Bello , et al., “Deleterious Impact of Donor‐Specific Anti‐HLA Antibodies Toward HLA‐Cw and HLA‐DP in Kidney Transplantation,” Transplantation 100, no. 1 (2016): 159–166, 10.1097/TP.0000000000000821. 26262501

[10] J. Visentin , T. Bachelet , O. Aubert , et al., “Reassessment of the Clinical Impact of Preformed Donor‐Specific Anti‐HLA‐Cw Antibodies in Kidney Transplantation,” American Journal of Transplantation 20, no. 5 (2020): 1365–1374, 10.1111/ajt.15766. 31883413

[11] A. Seitz , K. Mounsey , P. Hughes , et al., “Isolated Pre‐Existing HLA‐DP Donor‐Specific Antibodies Are Associated With Poorer Outcomes in Renal Transplantation,” Kidney International Reports 7, no. 10 (2022): 2251–2263, 10.1016/j.ekir.2022.07.014. 36217531 PMC9546735

[12] T. Laboux , R. Lenain , J. Visentin , et al., “Impact of Preformed Donor‐Specific Anti‐HLA‐Cw and Anti‐HLA‐DP Antibodies on Acute Antibody‐Mediated Rejection in Kidney Transplantation,” Transplant International 36 (2023): 11416, 10.3389/ti.2023.11416. 38076227 PMC10698113

[13] Q. Pan , Y. You , X. Wang , et al., “The Impact of Preformed and De Novo HLA‐DP Antibodies in Renal Transplantation, a Meta‐Analysis,” HLA 101, no. 2 (2023): 115–123, 10.1111/tan.14879. 36373504

[14] E. Mancebo , F. Diekmann , E. Palou , et al., “Spanish Guidelines for Kidney Transplantation in Highly Sensitized Patients With Donor‐Specific Anti‐HLA Antibodies,” Transplantation Reviews 39, no. 3 (2025): 100919, 10.1016/j.trre.2025.100919. 40209457

[15] R. Battle , D. Pritchard , S. Peacock , et al., “BSHI and BTS UK Guideline on the Detection of Alloantibodies in Solid Organ (And Islet) Transplantation,” International Journal of Immunogenetics 50, no. Suppl 2 (2023): 3–63, 10.1111/iji.12641. 37919251

[16] C. Usureau , R. Lhotte , V. Clichet , A. Foroutan , M. Devriese , and J. L. Taupin , “Refining cPRA Calculation to Improve HLA Compatibility Assessment in Organ Transplantation: A Detailed Picture of the Paris Waiting List,” HLA 104, no. 3 (2024): e15675, 10.1111/tan.15675. 39247974

[17] Agence de la biomédecine , Website, https://rams.agence‐biomedecine.fr/greffe‐renale‐0.

[18] L. Couzi , P. Malvezzi , L. Amrouche , et al., “Imlifidase for Kidney Transplantation of Highly Sensitized Patients With a Positive Crossmatch: The French Consensus Guidelines,” Transplant International 36 (2023): 11244, 10.3389/ti.2023.11244. 37448448 PMC10336835

[19] D. Bertrand , P. Gatault , C. Poulain , et al., “Sensitization and Deceased‐Donor Kidney Transplant Access in France,” Kidney International Reports 11, no. 4 (2026): 106340, 10.1016/j.ekir.2026.106340. 41853743 PMC12992927

[20] W. Manitpisitkul , C. Drachenberg , E. Ramos , et al., “Maintenance Immunosuppressive Agents as Risk Factors for BK Virus Nephropathy: A Case‐Control Study,” Transplantation 88 (2009): 83–88, 10.1097/TP.0b013e3181aa8d93. 19584685

[21] M. Ziemann , B. Suwelack , B. Banas , et al., “Determination of Unacceptable hla Antigen Mismatches in Kidney Transplant Recipients,” HLA 100, no. 1 (2022): 3–17, 10.1111/tan.14521. 34951119

[22] L. A. Baxter‐Lowe , A. Kucheryavaya , D. Tyan , and N. Reinsmoen , “CPRA for Allocation of Kidneys in the US: More Candidates ≥ 98% CPRA, Lower Positive Crossmatch Rates and Improved Transplant Rates for Sensitized Patients,” Human Immunology 77, no. 5 (2016): 395–402, 10.1016/j.humimm.2016.03.003. 27012168

