# Supplementary material for: Impact of Excluding Anti‐HLA‐C and ‐DP Antibodies From the Allocation System on Kidney Transplant Access
Source: HLA. 2026 Jul 7;108(1):e70822. doi: 10.1111/tan.70822 (PMC13339753; doi:10.1111/tan.70822)
Supplement: Supplementary file 1 — Figure S1: Association between relative loss of graft access and observed access to transplantation. Figure S2: Observed access to transplantation according to anti‐HLA‐C and anti‐HLA‐DP antibody profiles. Figure S3: Immunological donor offer refusal rates according to anti‐HLA‐C and anti‐HLA‐DP antibody profiles. [file TAN-108-e70822-s002.docx]

**Supplementary material**

**Supplementary Figure 1: Association between relative loss of graft access and observed access to transplantation**

(A) Cumulative incidence of kidney transplantation according to relative loss of graft access (RLGA) categories among patients with anti-HLA-C and/or anti-HLA-DP antibodies and classical cPRA < 100%. Patients were stratified into six RLGA categories (0%, 1–25%, 26–50%, 51–75%, 76–99%, and 100%). Curves were estimated using the Kaplan–Meier method, and differences between groups were assessed using the log-rank test.

**Supplementary Figure 2: Observed access to transplantation according to anti-HLA-C and anti-HLA-DP antibody profiles**

(A) Cumulative incidence of kidney transplantation in the whole cohort according to anti-HLA antibody specificity: no anti-HLA-C/DP antibodies (no αC/DP), anti-HLA-C antibodies only (αC), anti-HLA-DP antibodies only (αDP), and combined anti-HLA-C and anti-HLA-DP antibodies (αC+DP). Curves were estimated using the Kaplan–Meier method, and differences between groups were assessed using the log-rank test.

**Supplementary Figure 3: Multivariable analyses of factors associated with access to kidney transplantation** **according to anti-HLA-C and anti-HLA-DP antibody profiles**

Hazard ratios (HR) and 95% confidence intervals (CI) were obtained from a multivariable Cox proportional hazards model, including only variables with p < 0.05 in univariable analyses. A HR < 1 indicates a lower likelihood of receiving a transplant.

**Supplementary Figure 4: Immunological donor offer refusal rates according to anti-HLA-C and anti-HLA-DP antibody profiles**


(A) Immunological donor offer refusal rates according to antibody profile in patients without anti-HLA-C/DP antibodies (no C/DP), with isolated anti-HLA-C antibodies (C), isolated anti-HLA-DP antibodies (DP), or combined anti-HLA-C and anti-HLA-DP antibodies (C/DP).

Violin plots represent the distribution of immunological refusal rates in each group. Dashed horizontal lines indicate quartiles and median values. Group comparisons were performed using the Kruskal–Wallis test followed by Wilcoxon rank-sum tests with false discovery rate correction.
