# Supplementary figures and images for: Impact of Excluding Anti‐HLA‐C and ‐DP Antibodies From the Allocation System on Kidney Transplant Access
Source: HLA. 2026 Jul 7;108(1):e70822. doi: 10.1111/tan.70822 (PMC13339753; doi:10.1111/tan.70822)

Supplementary Figure 3  
A

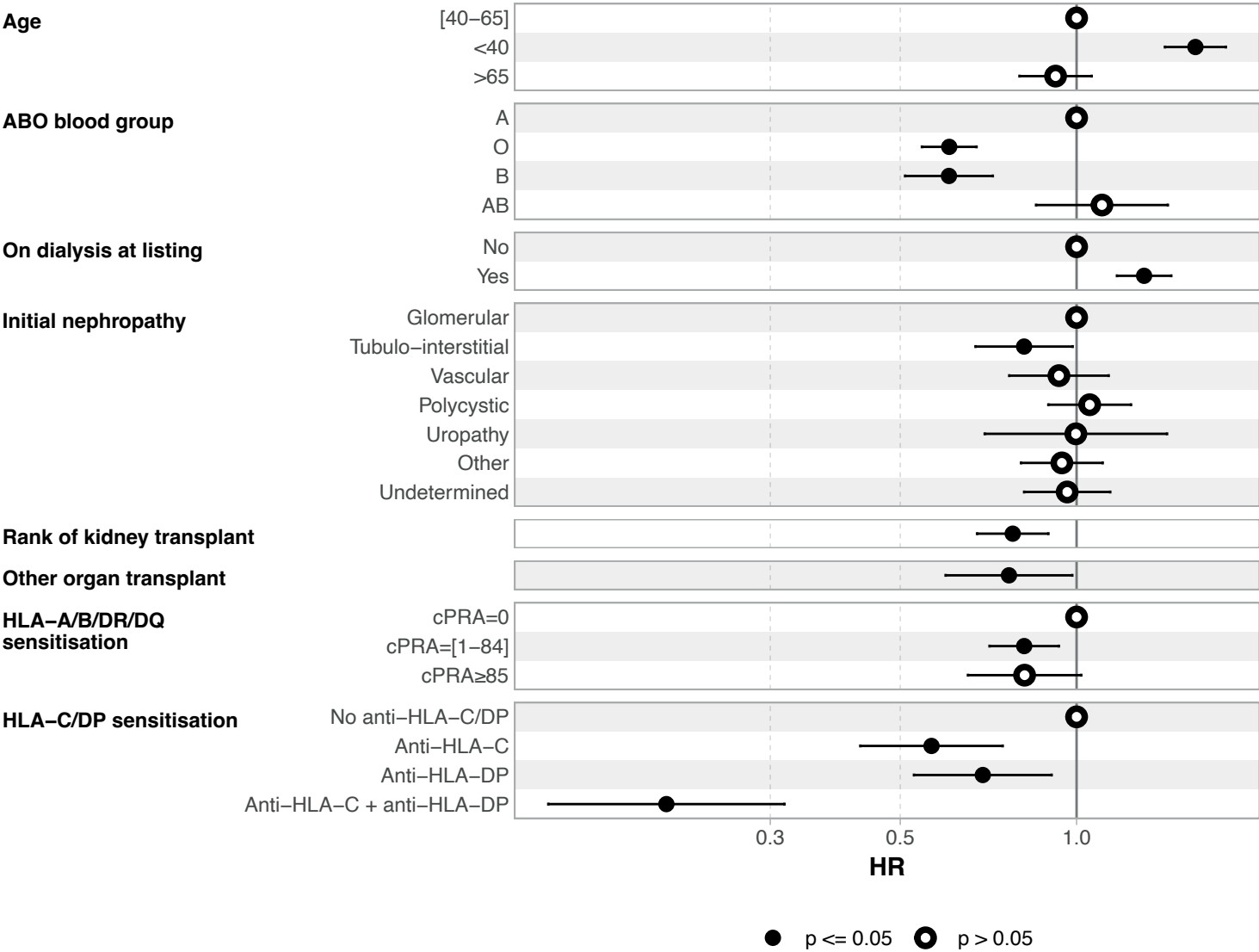

Supplement: Supplementary file 2 — Figure S4: Multivariable analyses of factors associated with access to kidney transplantation according to anti‐HLA‐C and anti‐HLA‐DP antibody profiles. [file TAN-108-e70822-s001.pdf]
